# FLOating-Window Projective Separator (FloWPS): A Data Trimming Tool for Support Vector Machines (SVM) to Improve Robustness of the Classifier

**DOI:** 10.3389/fgene.2018.00717

**Published:** 2019-01-15

**Authors:** Victor Tkachev, Maxim Sorokin, Artem Mescheryakov, Alexander Simonov, Andrew Garazha, Anton Buzdin, Ilya Muchnik, Nicolas Borisov

**Affiliations:** ^1^Department of Bioinformatics and Molecular Networks, OmicsWay Corporation, Walnut, CA, United States; ^2^Shemyakin-Ovchinnikov Institute of Bioorganic Chemistry, Moscow, Russia; ^3^Yandex N.V. Corporation, Moscow, Russia; ^4^I.M. Sechenov First Moscow State Medical University (Sechenov University), Moscow, Russia; ^5^Hill Center, Rutgers University, Piscataway, NJ, United States

**Keywords:** bioinformatics, machine learning, oncology, gene expression, support vector machines, personalized medicine

## Abstract

Here, we propose a heuristic technique of data trimming for SVM termed *FLOating Window Projective Separator* (*FloWPS*), tailored for personalized predictions based on molecular data. This procedure can operate with high throughput genetic datasets like gene expression or mutation profiles. Its application prevents SVM from extrapolation by excluding non-informative features. FloWPS requires training on the data for the individuals with known clinical outcomes to create a clinically relevant classifier. The genetic profiles linked with the outcomes are broken as usual into the training and validation datasets. The unique property of FloWPS is that irrelevant features in *validation* dataset that don’t have significant number of neighboring hits in the *training* dataset are removed from further analyses. Next, similarly to the *k* nearest neighbors (kNN) method, for each point of a *validation* dataset, FloWPS takes into account only the proximal points of the *training* dataset. Thus, for every point of a *validation* dataset, the *training* dataset is adjusted to form a *floating window*. FloWPS performance was tested on ten gene expression datasets for 992 cancer patients either responding or not on the different types of chemotherapy. We experimentally confirmed by leave-one-out cross-validation that FloWPS enables to significantly increase quality of a classifier built based on the classical SVM in most of the applications, particularly for polynomial kernels.

## Introduction

Support vector machine is one of the most popular machine learning methods in biomedical sciences with constantly growing impact and more than 11,000 citations in the PubMed-indexed literature^1^, of those ∼2,300 are only for the 2017 and first 6 months of 2018. This method has been successfully applied for a wide variety of biomedical applications like searching Dicer RNase cleavage sites on pre-miRNA (Ahmed et al., 2013), prediction of miRNA guide strands (Ahmed et al., 2009a), identification of poly(A) signals in genomic DNA (Ahmed et al., 2009b), finding conformational B-cell epitopes in antigens by nucleotide sequence (Ansari and Raghava, 2010). More recent developments include drug design according to physicochemical properties (Yosipof et al., 2018), learning on transcriptomic profiles for age recognition (Mamoshina et al., 2018), predictions of drug toxicities and other side effects (Zhang et al., 2018).

The performance quality of the classifiers based on these methods may reach the value of 0.80 or higher for the metrics such as ROC AUC^2^ and/or accuracy rate, e.g., for problems of age recognition (Mamoshina et al., 2018) and drug compound selection (Yosipof et al., 2018). However, although generally clearly helpful, the SVM approach frequently demonstrates insufficient performance in several applications for separating groups of the patients with different clinical outcomes (Mulligan et al., 2007; Ray and Zhang, 2009; Babaoglu et al., 2010; Kim et al., 2018). These failures were most likely caused by insufficient number of preceding clinical cases, which provokes overtraining of all machine learning algorithms. Particularly, the rareness of training points in the feature space leads to frequent extrapolations, and SVM method is known to be highly vulnerable to such conditions (Arimoto et al., 2005; Balabin and Lomakina, 2011; Balabin and Smirnov, 2012; Betrie et al., 2013).

In order to increase the performance of SVM for distinguishing between clinically relevant features, such as degrees of response to cancer therapies, we propose here a new method termed *FloWPS* for data trimming that generalizes the SVM technique by precluding extrapolation in the feature space. FloWPS acts by selecting for further analysis only those features that lay within the intervals of data projections from the training dataset. This approach can avoid extrapolations in favor of interpolations and thus increases a prediction quality of the output data. FloWPS combines somehow two methods, SVM and kNN (Altman, 1992), where kNN plays a particular role to extract informative features. The idea to combine feature extraction methods with SVM is well known (Tan and Gilbert, 2003; Kourou et al., 2015; Tan, 2016; Liu et al., 2017; Tarek et al., 2017). The approach proposed in this paper, however, is in principle a novelty, at least because its selection capacity is focused on every single point available for prediction.

We tested FloWPS on ten published gene expression datasets for totally 992 cancer patients treated with different types of chemotherapy with known clinical outcomes. In all the cases, the classifiers built using FloWPS outperformed standard SVM classifiers.

## Results

### Data Sources and Feature Selection

In this study, we investigated gene expression features associated with the responses to chemotherapy. The gene expression profiles were extracted from the datasets summarized in Table 1. The clinical outcome information was related to response on different chemotherapy regimens, linked with high throughput gene expression profiles for the individual patients.

**Table 1:** Clinically annotated gene expression datasets.

Reference	Dataset ID	Disease type	Treatment type	Experimental platform	Number of samples	Number of *core marker genes*
Hatzis et al., 2011; Itoh et al., 2014	GSE25066	Breast cancer with different hormonal and HER2 status	Neoadjuvant taxane + anthracycline	Affymetrix Human Genome U133 Array	235 (118 responders, 117 non-responders)	20
Horak et al., 2013	GSE41998	Breast cancer with different hormonal and HER2 status	Neoadjuvant doxorubicin + cyclophosphamide, followed by paclitaxel	Affymetrix Human Genome U133 Array	68 (34 responders, 34 non-responders)	11
Mulligan et al., 2007	GSE9782	Multiple myeloma	Bortezomib	Affymetrix Human Genome U133 Array	169 (85 responders, 84 non-responders)	18
Chauhan et al., 2012	GSE39754	Multiple myeloma	Vincristine + adriamycin + dexamethasone followed by ASCT	Affymetrix Human Exon 1.0 ST Array	124 (62 responders, 62 non-responders)	16
Terragna et al., 2016	GSE68871	Multiple myeloma	Bortezomib-thalidomide-dexamethasone (VTD)	Affymetrix Human Genome U133 Plus	98 (49 responders, 49 non-responders)	12
Amin et al., 2014	GSE55145	Multiple myeloma	Bortezomib followed by ASCT	Affymetrix Human Exon 1.0 ST Array	56 (28 responders, 28 non-responders)	14
Goldman et al., 2015; Walz et al., 2015	TARGET-50	Childhood kidney Wilms tumor	Vincristine sulfate + non-target drugs + conventional surgery + radiation therapy	Illumina HiSeq 2000	72 (36 responders, 36 non-responders)	14
Goldman et al., 2015; Tricoli et al., 2016	TARGET-10	Childhood B acute lymphoblastic leukemia	Vincristine sulfate + non-target drugs	Illumina HiSeq 2000	60 (30 responders, 30 non-responders)	14
Goldman et al., 2015	TARGET-20	Childhood acute myeloid leukemia	Non-target drugs including busulfan and cyclophosphamide	Illumina HiSeq 2000	46 (23 responders, 23 non-responders)	10
Goldman et al., 2015	TARGET-20	Childhood acute myeloid leukemia	Non-target drugs excluding busulfan and cyclophosphamide	Illumina HiSeq 2000	124 (62 responders, 62 non-responders)	16


Each patient was primarily labeled as either responder or non-responder to a treatment. For all the datasets taken from the GEO repository, we used the response criteria formulated in the respective original papers first publishing these data. Namely, for two breast cancer datasets, GSE25066 (Hatzis et al., 2011; Itoh et al., 2014) and GSE41998 (Horak et al., 2013), we considered *partial responders* as responders. For the first multiple myeloma dataset, GSE9782 (Mulligan et al., 2007), we took the (non)responder classification used by the authors, where patents with *complete* and *partial response* were annotated as responders, and with *no change* and *progressive disease* – as non-responders. For three other multiple myeloma datasets, GSE39753 (Chauhan et al., 2012), GSE68871 (Terragna et al., 2016), and GSE55145 (Amin et al., 2014), we considered *complete*, *near-complete* and *very good partial responders* as responders, whereas *partial*, *minor* and *worse* responders – as non-responders. For the datasets of pediatric Wilms kidney tumor, ALL and AML, extracted from the TARGET gene expression repository of National Cancer Institute (Goldman et al., 2015), the cases was classified according the distribution of the event-free survival time, which appeared to have two modes with different slopes (Supplementary Figure S1).

To preclude any possible bias that may affect the performance of machine-learning classifiers due to unequal representation of samples in two different classes (clinical responders and non-responders), numbers of responding and non-responding cases were equalized within each dataset. Equalization was done by taking the full *smaller* subset of those for the two classes (responders/non-responders), and then by random selection of samples from the *bigger* subset. Thus, each resulting dataset contained equal numbers of cases classified as responders and non-responders.

To engineer a plausible feature space, where the SVM can be applied efficiently, we proposed to select from tens of thousands of individual gene expression features only few of them, which produce a good separation of clinical responders from non-responders. To do so, for every dataset under investigation we selected its particular top 30 genes, whose expression levels taken one by one had the highest ROC AUC values for distinguishing responder and non-responder profiles. We made a number of top informative features equal to 30 because the usual number of samples in considered datasets was not lower than 50 (a direct heuristic number for degree of freedom). These *30 top marker genes*, and response statuses (100 for a responder, 0 for a non-responder) for all selected patients from all datasets are listed on Supplementary Table S1.

To produce more robust feature selection, for each dataset having, say, *N* samples, the leave-one-out procedure has been performed. Each individual sample was removed from the investigation one at a time, so *N* subdatasets each having *N*-1 individuals were generated. For each subdataset, the ROC AUC test was performed between responders and non-responders for each gene. The genes were next sorted according to their ROC AUC, and top 30 were selected for each subdataset. The final list of such *core informative* genes was generated as an intersection between top 30 selected genes for all *N* subdatasets. For every dataset under investigation, these final core sets are listed in Supplementary Table S2; the number of core marker genes is also shown on Table 1.

### Data Trimming for Application in SVM

We developed a first of its class data trimming^3^ tool termed FloWPS that has a potential to improve the performance of machine learning methods. Since extrapolation is a widely recognized Achilles heel of SVM (Arimoto et al., 2005; Balabin and Lomakina, 2011; Balabin and Smirnov, 2012; Betrie et al., 2013), FloWPS avoids it by using the rectangular projections along all irrelevant expression features that cause extrapolation during the SVM-based predictions for every validation point.

In this section we describe and investigate our data trimming procedure (FloWPS) as a preprocessing for SVM application.

Since the number of samples in most of the datasets used here was relatively low, we tested our classifier using the leave-one-out cross-validation method, which introduces lesser errors than the standard five-bin cross-validation scheme generally applied for bigger datasets. According to the leave-one-out approach, for each sample *i =* 1, *N* serves as a validation case whose response to the treatment had to be predicted, whereas all remaining samples, *j* = 1,…(*i*-1),(*i+*1)*,…,N*, collectively acts as a training dataset, and this procedure is repeated for all the samples. For machine leaning without data trimming, in a predefined feature space **F** = (*f*_1_,…, *f_s_* ) every sample *i*, given for the test, is assigned by a classifier, constructed to (*N*-1) samples used for training.

**FIGURE 1:**
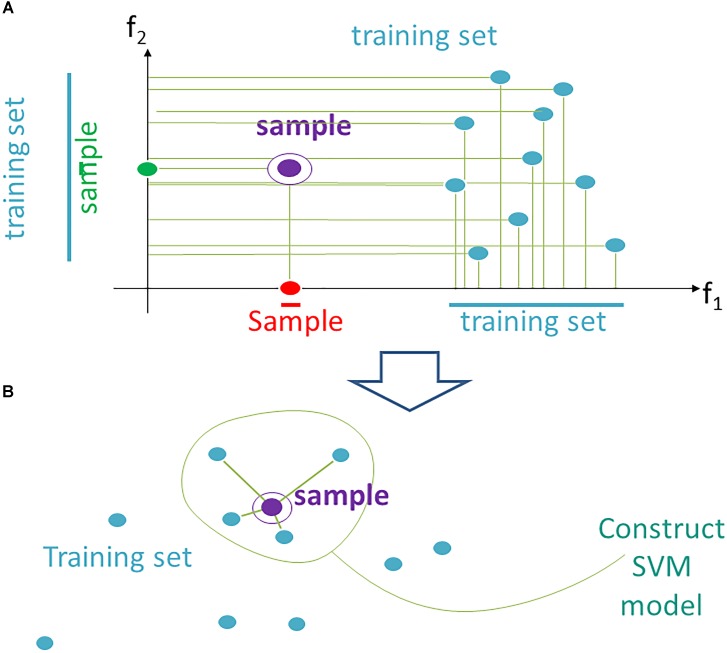
Data trimming pipeline. **(A)** selection of relevant features in FloWPS according to the *m*-condition. A violet dot shows the position of a validation point. Turquoise dots stand for the points from the training dataset. The features (here: *f_1_* and *f_2_*) are considered relevant when they satisfy the criterion that at least *m* flanking training points must be present on both sides relative to the validation point along the feature-specific axis. In the figure, it is exemplified that *m*-condition is satisfied for *f*_1_ feature when *m* = 0 only, and for the *f*_2_, when *m* ≤ 5. **(B)** After selection of the relevant features, only *k* nearest neighbors in the training sets are selected to construct the SVM model. On the figure, *k* = 4, although *k* starting from 20 was used in our calculations, to build SVM model.

According to the current data trimming approach, instead a fixed space **F** for all *N* testing samples, we propose using an individual space **F***_i_*, which contains individually adapted training data (of *N*-1 samples) for the testing sample *i*. It can be implemented using the following heuristics (Figure 1).

(1) From the whole predefined feature space **F** = (*f*_1_,…, *f*_s_ ) we extract a subset **F***_i_* (*m*), where *m* is a parameter. A feature *f_j_* is kept in **F***_i_*(*m*) if on its axis there are at least *m* projections from training samples, which are larger than *f_j_* (*i*), and, at the same time, at least *m*, which are smaller than *f_j_* (*i*). The procedure for extraction of a subset **F***_i_*(*m*) is illustrated in Figure 1A for a two-dimensional space **F** = (*f*_1_, *f*_2_). A violet point stands for the validation sample in the feature space. Turquoise dots represent scattering of the training points. For example, the *m*-condition for the feature *f*_2_ is satisfied when *m* = 0,1,2,3,4,5 (projection of the training set on *f*_2_ axis has five points both below and above the validation point), whereas for the feature *f*_1_ it is satisfied only for *m* = 0 (projection of the validation point on axis *f*_1_ lies outside of the cloud of training points).

(2) In **F***_i_* (*m*) we keep for training only *k* closest samples (from given (*N*-1) samples); *k* is also a parameter (Figure 1B; note that although for the sake of simplicity *k* = 4 in the picture, in the computational trials we varied *k* from 20 to *N*-1).

**FIGURE 2:**
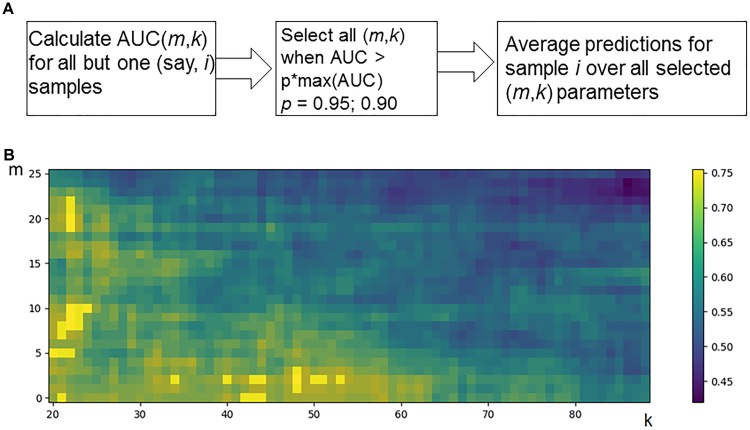
Optimization of data trimming parameters *m* and *k* for a given individual. **(A)** Overall scheme for prediction for an individual sample *i* = 1, *N*. All but one individuals serve as a training dataset. For a training dataset at the fitting step, the AUC for a classifier prediction is calculated and plotted **(B)** as a function of data trimming parameters *m* and *k*. Positions of this AUC topogram where AUC > *p* ⋅ max(AUC), *p* = 0.95, are considered *prediction-accountable* (highlighted with bright yellow color) and form the prediction-accountable set *S*. This AUC topogram, as well as the set *S*, is individual for every validation point *i*.

Hence, for every individual *i =* 1, *N*, and *m* and *k* parameter values, the predicted classification values are obtained [i.e., *predictions* *P_i_* (*m*,*k*)*, i =* 1, *N*]. Considering known response status for each sample *i*, it is possible to calculate AUC values for a whole set of samples as a function over whole range of the parameters *m* and *k* (Figure 2B). Since the predicted classification efficiencies depend upon the chosen values for *m* and *k*, it is possible to interrogate the AUC values over the full lattice of all possible (*m*, *k*) pairs.

We propose an algorithm of achieving the optimal (*m*,*k*)-settings for a final classifier (Figure 2A). The AUC threshold (θ) is set to θ* = p* ⋅ max(AUC), where max(AUC) is the maximal value of AUC, taken over the set of all possible (*m*, *k*) pairs, and the parameter *p* equals to a user-defined confidence threshold. To illustrate performance of this approach, we took two alternative values of *p* = 0.95 or 0.90, and then considered all the (*m*,*k*) pair positions on the AUC(*m*,*k*) topogram. We next screened for the positions where AUC exceeded the threshold θ, and the total combination of these positions was taken as the *prediction-accountable set S* (Figure 2B; prediction-accountable positions are shown in yellow). The final prediction of FloWPS (*P_F_*) for a certain validation case should be calculated by averaging the SVM predictions, *P*(*m,k*), over the whole set of positions belonging to the prediction-accountable set *S*, according to the formula: *P_F_* = *mean_S_*(*P*(*m,k*)).

The usual SVM method, i.e., without FloWPS data trimming, corresponds to a very right and bottom corner of the AUC(*m*,*k*) topogram (Figure 2B), with the parameter settings *m* = 0, *k* = *N* - 1. On the example shown in Figure 2B, the classical SVM, without any doubt, provides essentially lower accuracy than FloWPS.

### FloWPS Performance for Default SVM Settings

**FIGURE 3:**
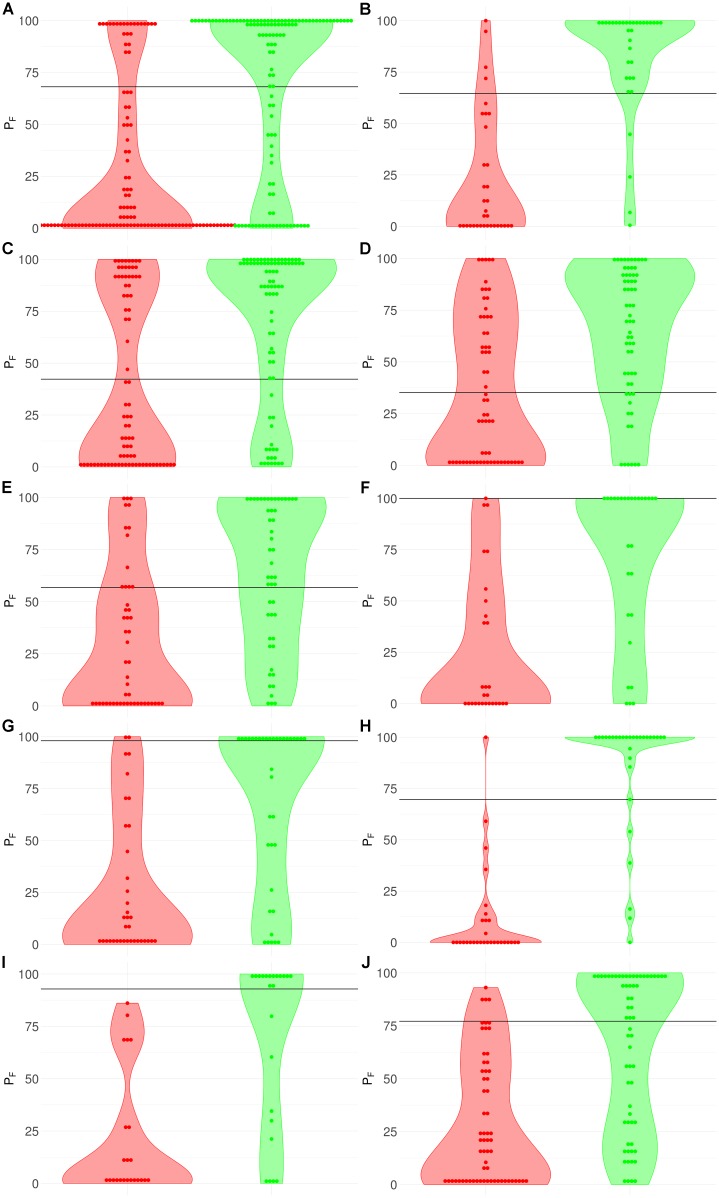
Distribution (violin plots together with each instance showed as a red/green dot) of FloWPS predictions (*P_F_*) for patients without (red plots and dots) and with (green plots and dots) positive clinical response to chemotherapy treatment. For FloWPS, *core marker genes* and *p* = 0.90 settings were used. Black horizontal line shows the discrimination threshold (τ) between responders and non-responders for each classifier. Panels represent different data sources, **(A)** GSE25066; **(B)** GSE41998; **(C)** GSE9782; **(D)** GSE39754; **(E)** GSE68871; **(F)** GSE55134; **(G)** TARGET-50; **(H)** TARGET-10; **(I)** and **(J)**: TARGET-20 with and without busulfan and cyclophosphamide, respectively.

**FIGURE 4:**
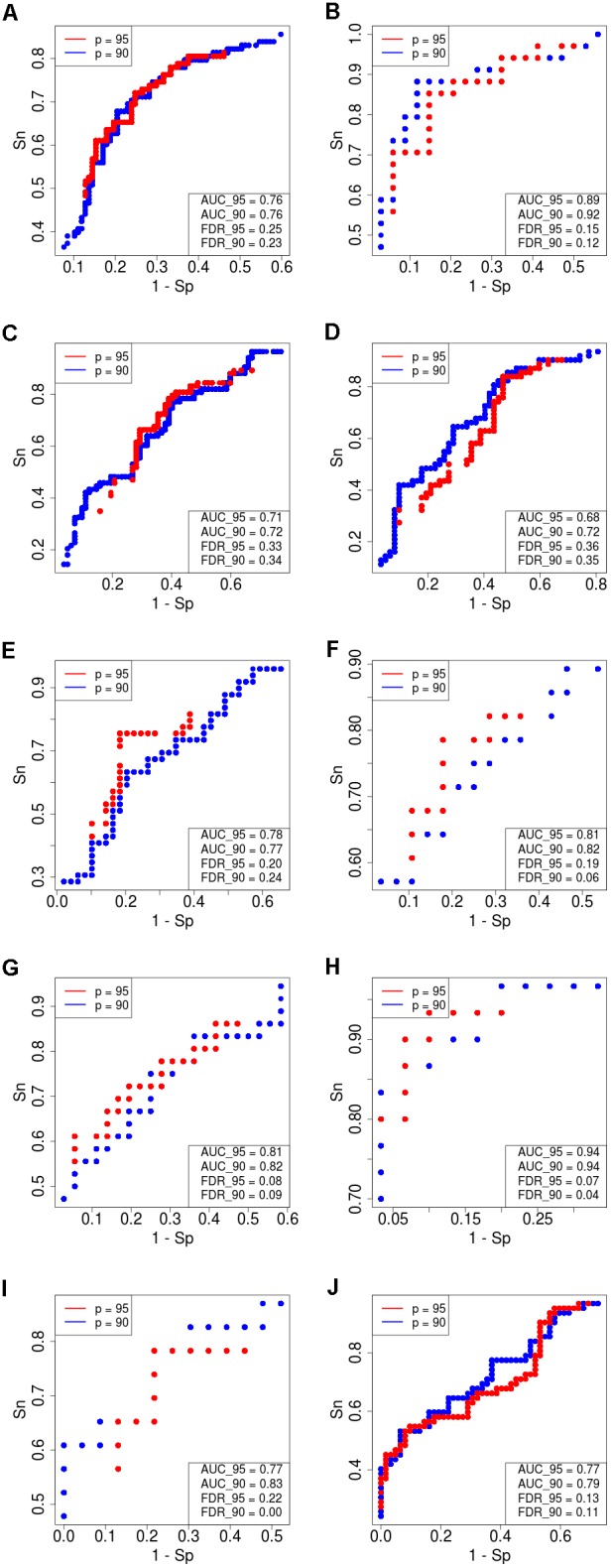
Receiver–operator curves (ROC) showing the dependence of sensitivity (*Sn*) upon specificity (*Sp*) for FloWPS-based classifier of treatment response for datasets with *core marker genes*. Red dots: confidence parameter *p* = 0.95, blue dots: *p* = 0.90. Panels represent different clinically annotated datasets, **(A)** GSE25066; **(B)** GSE41998; **(C)** GSE9782; **(D)** GSE39754; **(E)** GSE68871; **(F)** GSE55134; **(G)** TARGET-50; **(H)** TARGET-10; **(I,J)** TARGET-20 with and without busulfan and cyclophosphamide, respectively.

At first, we investigated performance of FloWPS on ten cancer gene expression datasets (Table 1) with the default SVM settings (linear kernel and cost/penalty parameter *C* = 1). During our calculations, the FloWPS classifier was first fitted for the training dataset without a sample (say, *i*) to be classified. For these all (*N*-1) samples AUC*_i_*(*m*,*k*) was calculated as a function of data trimming parameters *m* and *k* (see Figure 2A). This enabled finding the prediction-accountable set *S_i_* in the AUC*_i_*(*m*,*k*) topogram (on Figure 2B, the set was marked with bright yellow). The *m* and *k* values from the set *S_i_* were then used for data trimming and classifying of a single sample *i*. In parallel, we applied the standard SVM algorithm for leave-one-out cross-validation without data trimming, i.e., *m* = 0, *k* = *N*-1 for each training sub-dataset. The comparison is shown on Table 2, Supplementary Table S3, and Figures 3, 4.

**Table 2:** Performance of clinical response classifiers for clinically annotated gene expression datasets.

Dataset	Top 30 marker genes						Core marker genes					
	SVM		FloWPS *p* = 0.95		FloWPS *p* = 0.90		SVM		FloWPS *p* = 0.95		FloWPS *p* = 0.90	
						
	AUC	FDR	AUC	FDR	AUC	FDR	AUC	FDR	AUC	FDR	AUC	FDR
GSE25066 (Hatzis et al., 2011; Itoh et al., 2014)	**0.70**	0.28	**0.76**	0.10	**0.77**	0.13	**0.73**	0.26	**0.76**	0.25	**0.76**	0.23
GSE41998 (Horak et al., 2013)	**0.79**	0.25	**0.87**	0.14	**0.91**	0.14	**0.87**	0.14	**0.89**	0.15	**0.92**	0.12
GSE9782 (Mulligan et al., 2007)	**0.73**	0.28	**0.78**	0.22	**0.76**	0.17	**0.68**	0.33	**0.71**	0.33	**0.72**	0.34
GSE39754 (Chauhan et al., 2012)	**0.65**	0.36	**0.68**	0.27	**0.71**	0.34	**0.65**	0.36	**0.68**	0.36	**0.72**	0.35
GSE68871 (Terragna et al., 2016)	**0.66**	0.35	**0.75**	0.25	**0.74**	0.27	**0.68**	0.33	**0.78**	0.20	**0.77**	0.24
GSE55145 (Amin et al., 2014)	**0.84**	0.19	**0.86**	0.11	**0.90**	0.11	**0.77**	0.24	**0.81**	0.19	**0.82**	0.06
TARGET-50 (Goldman et al., 2015; Walz et al., 2015)	**0.64**	0.35	**0.75**	0.13	**0.78**	0.16	**0.72**	0.26	**0.81**	0.08	**0.82**	0.09
TARGET-10 (Goldman et al., 2015; Tricoli et al., 2016)	**0.85**	0.16	**0.86**	0.14	**0.87**	0.12	**0.87**	0.13	**0.94**	0.07	**0.94**	0.04
TARGET-20 (Goldman et al., 2015) with busulfan and cyclophosphamide	**0.74**	0.26	**0.79**	0.16	**0.79**	0.17	**0.76**	0.23	**0.77**	0.22	**0.83**	0.00
TARGET-20 (Goldman et al., 2015) w/o busulfan and cyclophosphamide	**0.73**	0.28	**0.76**	0.30	**0.76**	0.27	**0.74**	0.26	**0.77**	0.13	**0.79**	0.11


*Area-under-curve (AUC) and false discovery rate (FDR) values calculated for each version of a classifier are shown. All calculations were made using leave-one-out cross-validation approach.*

The discrimination threshold (τ), which is shown as a black horizontal line on Figure 3 (so that any sample with FloWPS prediction value above τ is classified as a responder, and below it – as a non-responder), was set to minimize the sum of FP and FN predictions.

For every dataset, confidence parameter *p* and scheme of gene selection, FloWPS classifier demonstrated the ROC AUC exceeding the corresponding value for the classical SVM (Table 2). For three datasets out of ten, AUC for classical SVM was between 0.64 and 0.68. For all these cases, application of FloWPS with confidence level *p* = 0.90 enabled obtaining essentially better AUC values ranging between 0.71 and 0.78.

The comparison of classifier’s quality by another metric, the FDR^4^, has demonstrated similar results: FDR was lower for FloWPS than for classical SVM for almost all the cases (Table 2, columns without boldface font). Other metrics, such as sensitivity (Sn), specificity (Sp), accuracy rate (ACC) and MCC^5^ also strongly tend to be higher for FloWPS than for classical SVM without data trimming (Supplementary Table S3).

### FloWPS Performance at Different Settings and Comparison With Alternative Data Reduction Approach

**FIGURE 5:**
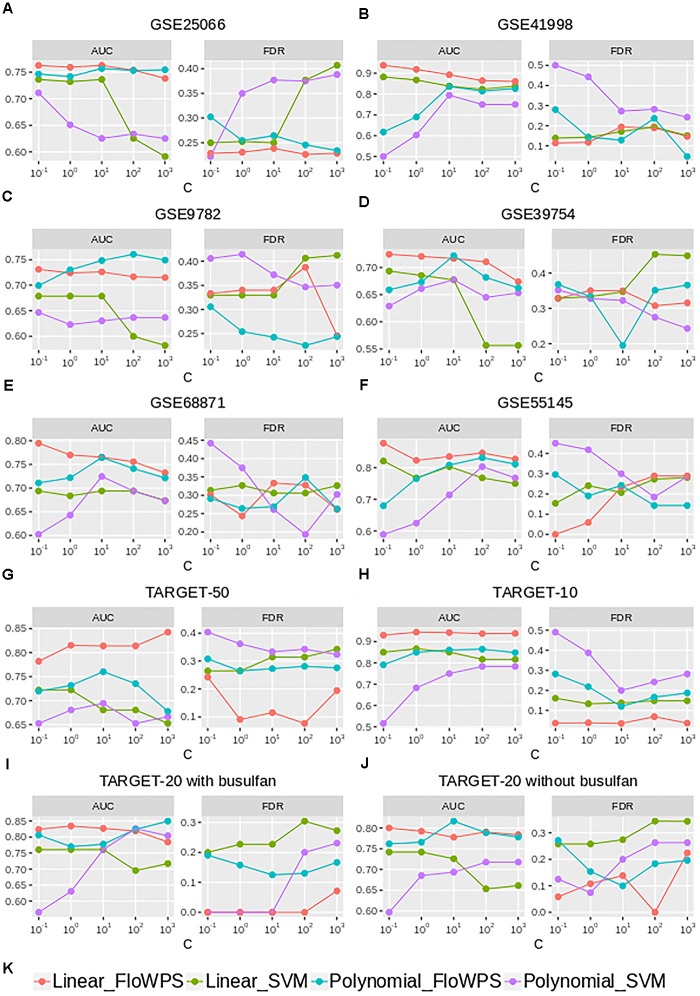
AUC and FDR for (non)responders classifier as a function of cost/penalty parameter *C* for classical SVM (without data trimming) and FloWPS for both linear and polynomial kernels. Calculations were done for core marker gene datasets and confidence parameter *p* = 0.90. Different panels represent different datasets, **(A)** GSE25066; **(B)** GSE41998; **(C)** GSE9782; **(D)** GSE39754; **(E)** GSE68871; **(F)** GSE55134; **(G)** TARGET-50; **(H)** TARGET-10; **(I,J)** TARGET-20 with and without busulfan and cyclophosphamide, respectively. **(K)** Legend showing FloWPS and SVM modifications.

Although the classifier quality tended to be higher for data trimming than for default SVM settings, the advantages were different in different cancer datasets. The FloWPS performance, therefore, was investigated for different SVM kernels (linear vs. polynomial) and different values for cost/penalty parameters *C* (ranged from 0.1 to 1000), Figure 5 and Supplementary Table S4. These calculations were done for the core marker gene datasets and FloWPS confidence parameter *p* = 0.90. The advantage of FloWPS over SVM is more essential in the conditions vulnerable to SVM overtraining, e.g., for linear kernel with high values of the cost/penalty parameter (C = 100 or 1000) or for polynomial kernel, where SVM may be easily overfitted. Fortunately, FloWPS precludes such overfitting, thus raising AUC and decreasing FDR. The same pattern was also seen for the Sn, Sp, ACC and MCC values (Supplementary Table S4).

Note that FloWPS is not the only possible data reduction/feature selection method, which may be used for preprocessing to improve the classifier’s quality. To try a simple alternative to FloWPS, which is, however, not specific to individual samples, we did calculations based on PCA mode rather than original features. The number of PCs taken for building the SVM model, may act as a parameter, which is optimized in a manner similar to optimization of *m* and *k* for FloWPS. Namely, a maximum for AUC as a function of PC number is found and then used as the optimal number of PCs for an SVM-based prediction.

Thus, we compared the classifier qualities for three methods, namely classical SVM without data reduction, PCA-assisted SVM with pre-trained PC number, and FloWPS with the confidence parameter *p* = 0.90 (Table 3; note that both classical SVM and FloWPS calculations were done using gene expression features rather than PCs). The calculations were done for core marker gene datasets and cost/penalty SVM parameters *C* = 1 and 100. For linear kernel, several datasets had comparable AUC for simple PCA-assisted data reduction and for FloWPS (Table 3). However, for polynomial kernel FloWPS essentially outperformed the PCA-assisted data reduction, most likely due to bigger risk of overtraining for SVM with nonlinear kernels.

**Table 3:** AUC of (non)responder classifier for classical SVM without data reduction (SVM), PCA-assisted SVM (PCA) and FloWPS with confidence parameter *p* = 0.90.

Dataset	Linear kernel						Polynomial kernel					
	*C* = 1			*C* = 100			*C* = 1			*C* = 100		
				
	SVM	PCA	FloWPS	SVM	PCA	FloWPS	SVM	PCA	FloWPS	SVM	PCA	FloWPS
GSE25066 (Hatzis et al., 2011; Itoh et al., 2014)	0.73	0.77	0.76	0.63	0.77	0.75	0.65	0.67	0.74	0.63	0.66	0.75
GSE41998 (Horak et al., 2013)	0.87	0.84	0.92	0.82	0.88	0.86	0.60	0.62	0.69	0.75	0.74	0.81
GSE9782 (Mulligan et al., 2007)	0.68	0.72	0.72	0.60	0.72	0.72	0.62	0.68	0.73	0.64	0.68	0.76
GSE39754 (Chauhan et al., 2012)	0.69	0.68	0.72	0.56	0.68	0.71	0.66	0.61	0.67	0.65	0.61	0.68
GSE68871 (Terragna et al., 2016)	0.68	0.68	0.77	0.69	0.68	0.76	0.64	0.65	0.72	0.69	0.76	0.74
GSE55145 (Amin et al., 2014)	0.77	0.84	0.82	0.77	0.84	0.85	0.63	0.73	0.77	0.80	0.73	0.83
TARGET-50 (Goldman et al., 2015; Walz et al., 2015)	0.72	0.75	0.82	0.68	0.76	0.81	0.68	0.64	0.73	0.65	0.72	0.74
TARGET-10 (Goldman et al., 2015; Tricoli et al., 2016)	0.87	0.85	0.94	0.82	0.83	0.94	0.68	0.65	0.85	0.78	0.83	0.86
TARGET-20 (Goldman et al., 2015) with busulfan and cyclophosphamide	0.76	0.78	0.83	0.70	0.80	0.82	0.63	0.63	0.77	0.83	0.72	0.82
TARGET-20 (Goldman et al., 2015) w/o busulfan and cyclophosphamide	0.74	0.81	0.79	0.65	0.79	0.79	0.69	0.68	0.77	0.72	0.69	0.79


## Discussion

It was seen previously that SVM sometimes fails when it is intended for distinguishing fine biomedical properties such as disease progression prognosis or assessment of clinical efficiency of drugs for an individual patient, using high throughput molecular data, e.g., complete DNA mutation or gene expression profiles (Ray and Zhang, 2009; Babaoglu et al., 2010). Particularly, for many biologically relevant applications, SVM occurred either fully incapable to predict drug sensitivity (Turki and Wei, 2016), or demonstrated poorer performance than competing method for machine learning (Davoudi et al., 2017; Cho et al., 2018; Jeong et al., 2018; Leite et al., 2018; Sauer et al., 2018; Yosipof et al., 2018). Thus, the tool for improvement of SVM performance is certainly needed.

In this study, we investigated ten sets of gene expression data for cancer patients treated with different anti-cancer drugs with known clinical outcomes, where the original dimension of samples (patients) is many hundreds times larger than the numbers of patients. So, the first problem in such applications was to extract an appropriate number of features, in which space one could achieve a classifier-predictor with a high level of quality. There are many authors focused to resolve the preprocessing problem (Tan and Gilbert, 2003; Kourou et al., 2015; Tan, 2016; Liu et al., 2017; Tarek et al., 2017). Some feature selection methods, like the DWFS wrapping tool (Soufan et al., 2015), use sophisticatedly designed approaches such as genetic algorithms to improve the classifier quality. In this paper we proposed one more, FloWPS, which is very different from all known. Its critical characteristic is that for every single new sample, which class has to be predicted, the method extracted its individual sub-space and, more, in that subspace takes for training data an appropriate subset of samples.

**FIGURE 6:**
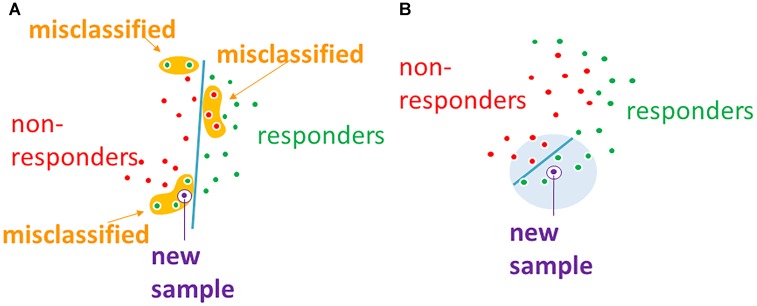
**(A)** Global machine learning methods, such as SVM, may fail to separate classes in datasets without global order. **(B)** Machine-learning with data trimming works locally and may separate classes more accurately.

FloWPS data trimming method simultaneously combines the advantages of both *global* (like SVM) and local (like kNN) (Altman, 1992) methods of machine learning, and successfully acts even when purely local and global approaches fail. The failure of SVM, which we have observed at least for 3 out of 10 datasets in the current study (Table 2), means that there is no strict *distant order* in the placement of responder and non-responder points in the space of gene expression features. Yet, the lack of *distant* order does not necessary mean the absence of *local* order (Figure 6). The latter may be detected using *local* methods such as kNN, which has been confirmed by our FloWPS (Table 2 and Figures 3, 5). The FloWPS advantages are better seen for SVM with polynomial than for linear kernel due to higher risk of overtraining on such models (Figure 5 and Table 3).

We hypothesize that FloWPS and data trimming may be also helpful for improving other learning methods based on multi-omics data, including nowadays-flourishing deep learning approaches (Bengio et al., 2013; LeCun et al., 2015; Schmidhuber, 2015).

## Materials and Methods

### Preprocessing of Gene Expression Data

For the datasets investigated using the Affymetrix microarray hybridization platforms, gene expression data were taken from the series matrices deposited in the GEO public repository and then quantile-normalized (Bolstad et al., 2003) using the R package *preprocessCore* (Bolstad, 2018). All pediatric datasets taken from the TARGET database (Goldman et al., 2015) contained results of NGS mRNA profiling at Illumina HiSeq 2000 platforms; they were normalized using R package *DESeq2* (Love et al., 2014).

### SVM Calculations

All the SVM calculations with linear and polynomial kernels were performed using the Python package *sklearn* (Pedregosa et al., 2012) that employs the C++ library ‘libsvm’ (Chang and Lin, 2011). The penalty parameter *C* varied from 0.1 to 1000 for different calculations. Other SVM parameters had the default settings for the *sklearn* package.

### Plot Preparations

AUC(*m*,*k*) topograms, like Figure 2B, were plotted using *mathplotlib* Python library (Hunter, 2007). Violin plots for FloWPS predictions (see Figure 3) for responders and non-responders were plotted using the *ggplot2* R package (Wilkinson, 2011).

## Availability of Data and Materials

The datasets analyzed during the current study are available in the GEO repository,

https://www.ncbi.nlm.nih.gov/geo/query/acc.cgi?acc=GSE25066

https://www.ncbi.nlm.nih.gov/geo/query/acc.cgi?acc=GSE41998

https://www.ncbi.nlm.nih.gov/geo/query/acc.cgi?acc=GSE9782

https://www.ncbi.nlm.nih.gov/geo/query/acc.cgi?acc=GSE39754

https://www.ncbi.nlm.nih.gov/geo/query/acc.cgi?acc=GSE68871

https://www.ncbi.nlm.nih.gov/geo/query/acc.cgi?acc=GSE55145

ftp://caftpd.nci.nih.gov/pub/OCG-DCC/TARGET/WT/mRNA-seq/

ftp://caftpd.nci.nih.gov/pub/OCG-DCC/TARGET/AML/mRNA-seq/

ftp://caftpd.nci.nih.gov/pub/OCG-DCC/TARGET/ALL/mRNA-seq/

The Python module that performs data trimming according to the FloWPS method for different values of parameters *m* and *k*, as well as the R code that makes FloWPS predictions using the results obtained with the Python module, and a README manual how to use these codes, were deposited on Gitlab and are available by the link: https://gitlab.com/oncobox/flowps.

## Ethics Statement

Current research did not involve any new human material. All the gene expression data that were used for research, were taken from publicly available repositories Gene Expression Omnibus (GEO) and TARGET, and had been previously anonymized by the teams, who had worked with them.

## Author Contributions

NB designed the overall research, suggested the principles of data trimming and prediction-accountable set, and wrote most parts of the manuscript. VT performed most part of calculations. MS suggested datasets with clinical responders and non-responders and performed feature selection. AM wrote the initial version of computational code. AS adapted this code for parallel calculations. AG tested and debugged the computational code. IM and AB essentially improved the manuscript upon the draft version has been prepared. AB preformed the overall scientific supervision of the project.

## Conflict of Interest Statement

VT, MS, AS, AG, AB, and NB were employed by OmicsWay Corporation, Walnut, CA, United States. AM was employed by Yandex N.V. Corporation, Moscow, Russia. The remaining author declares that the research was conducted in the absence of any commercial or financial relationships that could be construed as a potential conflict of interest.

## Footnotes

1 This is the result of a PubMed query https://www.ncbi.nlm.nih.gov/pubmed/?term=support+vector+machine_

2 The ROC (receiver–operator curve) is a widely-used graphical plot that illustrates the diagnostic ability of a binary classifier system as its discrimination threshold is varied. The ROC is created by plotting the true positive rate (TPR) against the false positive rate (FPR) at various threshold settings. The area under the ROC curve, called ROC AUC, or simply AUC, is routinely employed for assessment of any classifier’s quality.

3 Data trimming is the process of removing or excluding extreme values, or outliers, from a dataset (Turkiewicz, 2017).

4 FDR shows the percentage of *false positive* (FP) predictions among all those classified as positive, FDR = FP/(FP + TP), where TP means *true positive*.

5 MCC can be calculated from the confusion matrix, $MCC=\frac{TP\cdot TN-FP\cdot FN}{\sqrt{\left(TP+FP\right)\left(TP+FN\right)\left(TN+FP\right)\left(TN+FN\right)}}$

## Abbreviations

ALL: acute lymphoblastic leukemia
AML: acute myelogenous leukemia
ASCT: allogeneic stem cell transplantation
AUC: area under curve
FDR: false discovery rate
FloWPS: floating window projective separator
FP: false positive
FN: false negative
GEO: gene expression omnibus
GSE: GEO series
HER2: human epidermal growth factor receptor 2
kNN: *k* nearest neighbors
MCC: Matthews correlation coefficient
mRNA: messenger ribonucleic acid
NGS: next-generation sequencing
PC: principal component
PCA: principal component analysis
ROC: receiver operating characteristic
SVM: support vector machine
TN: true negative
TP: true positive
VTD: velcade, thalidomide and dexamethasone
