# Supplementary material for: FLOating-Window Projective Separator (FloWPS): A Data Trimming Tool for Support Vector Machines (SVM) to Improve Robustness of the Classifier
Source: Front Genet. 2019 Jan 15;9:717. doi: 10.3389/fgene.2018.00717 (PMC6341065; doi:10.3389/fgene.2018.00717)
Supplement: Supplementary file 5 [file Data_Sheet_1.docx]

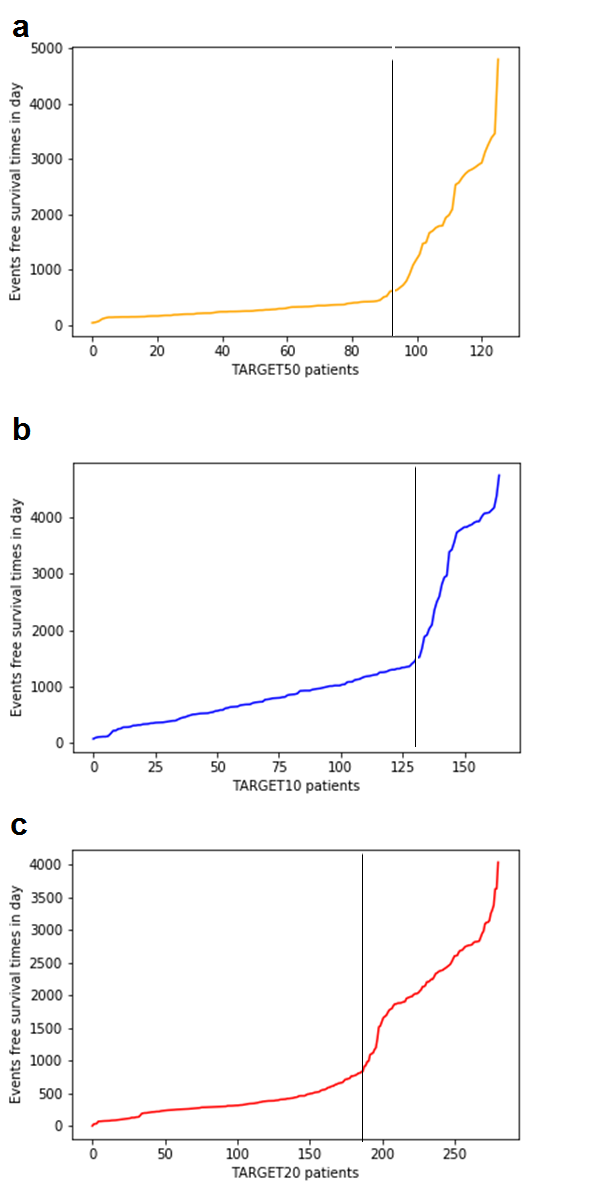


Supplementary figure S1. Distribution of event-free time for the patients of TARGET-50 (**a**), TARGET-10 (**b**) and TARGET-20 (**c**) datasets (Goldman et al., 2015). Patients to the left from vertical line were considered as non-responders, and to the right from this line – as responders to the treatment.

**References**

Goldman, M., Craft, B., Swatloski, T., Cline, M., Morozova, O., Diekhans, M., et al. (2015). The UCSC Cancer Genomics Browser: update 2015. *Nucleic Acids Res.* 43, D812–D817. doi:10.1093/nar/gku1073.
